# Recent advances in Bayesian inference of isolation-with-migration models

**DOI:** 10.5808/GI.2019.17.4.e37

**Published:** 2019-11-13

**Authors:** Yujin Chung

**Affiliations:** Department of Applied Statistics, Kyonggi University, Suwon 16227, Korea

**Keywords:** bayesian analysis, coalescent theory, gene flow, isolation-with-migration model, phylogeny

## Abstract

Isolation-with-migration (IM) models have become popular for explaining population divergence in the presence of migrations. Bayesian methods are commonly used to estimate IM models, but they are limited to small data analysis or simple model inference. Recently three methods, IMa3, MIST, and AIM, resolved these limitations. Here, we describe the major problems addressed by these three software and compare differences among their inference methods, despite their use of the same standard likelihood function.

## Introduction

**Fig. 1.**
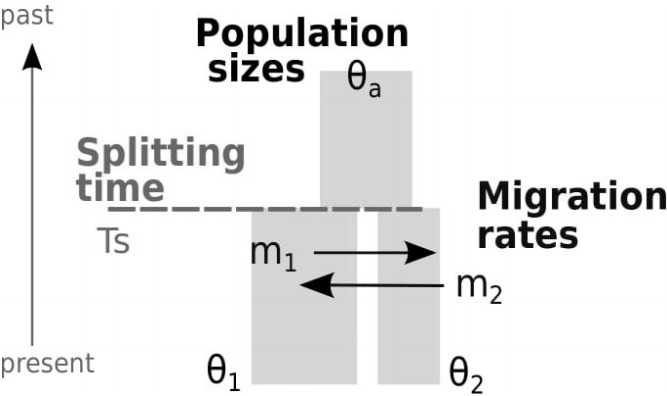
Isolation-with-migration model with six demographic parameters.

Divergence between populations and species has been a major interest in population genetics and evolution. Estimating divergence from genetic data is difficult because of conflicting evolutionary processes. Genetic drift elevates divergence between populations or between species, while gene flow can remove signals of divergence [1]. An isolation-with-migration (IM) model is a widely used demographic model describing the two conflicting signals. A typical 2-population IM model with six parameters (Fig. 1) depicts two populations (sizes θ_1_ and θ_2_, respectively) that arise from a single ancestral population (size θ_a_) at time *T_S_* in the past, while the two populations may exchange migrants at rates m_1_ and m_2_ ([1-3] for notations). Both population sizes and migration rates are assumed to be constant over time [4].

The challenges of inferring isolation models (with *no migrations*) and even phylogeny have been addressed by using a multispecies coalescent framework [5-11]. However, ignoring migrations can result in a biased estimation of splitting times of populations/species and may lead to a wrong phylogenetic tree estimation [12-16]. Efforts to distinguish between isolation and migration began about 20 years ago, and many methods have employed a Markov chain Monte Carlo (MCMC) simulation to infer an IM model [2-4,17-21]. However, most methods have a major roadblock of a long computational time of an MCMC simulation, which typically limits the amount of data that can be analyzed [1]. In addition, the joint estimation of both phylogeny and an IM model is known to be tremendously difficult [16].

Recently, three methods have been developed to address the scalability of the data and/or to jointly infer phylogeny in the presence of gene flow. IMa3 [16] is the most recent version of IM/IMa series software and infers the phylogeny and IM models. MIST [1] needs a known (or assumed) phylogeny but is able to analyze thousands of loci. AIM [14,22] is a package in the popular BEAST platform and also infers phylogeny in the presence of gene flow. Similar to other methods, these three methods implement the standard probabilistic framework and employ an MCMC simulation for inference.

The advent of many inference methods is not commensurate to our skills of analysis using those programs. In order to ensure the use of appropriate programs and to correctly interpret results, it is essential to understand the inference methods used and the results that the programs provide. IMa3, MIST, and AIM all use similar standard probabilistic models and apply Bayesian inference, but their inference strategies and the types of results may be different. Therefore, users must first understand the differences in their inference methods.

To elucidate the current state of the art in the analysis of IM models, in this review article, we compare the three methods and accompanying software, IMa3, MIST, and AIM (BEAST platform). In particular, the data type and the underlying model structures will be discussed, followed by a brief summary of an MCMC algorithm and mixing issue. Then, this review article will focus on comparison of the advanced methods: IMa3, MIST, and AIM. We do not intend to explain the basic concepts of standard probabilistic models and MCMC algorithms, but extensive reviews of them are available elsewhere [9,23-25].

## DNA Alignments

One of the most common types of data used in the analysis of IM models and phylogeny is DNA sequence alignments. Most methods, including IMa3, MIST, and AIM, assume the alignments are correct, although they are estimated from models of insertions and deletions [26]. The relatedness of homologous DNA sequences is considered to the result from past branching processes, so the DNA sequence alignments must be orthologs [22]. Moreover, no selection but a neutral evolution is assumed to act on alignments. Since most methods typically assume that there is no recombination within a locus and free recombination between loci, alignments should not overlap or be closely located. Moreover, filtering using a four-gamete test [27] is essential to minimize potential recombination within a locus.

## Standard Model Structure

**Fig. 2.**
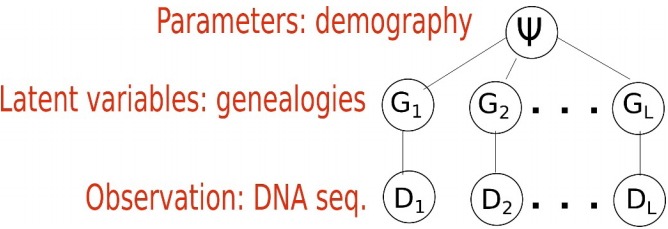
Standard model structure. Each locus has its own genealogy. Given genealogy, the genetic data and demography are assumed to be independent.

When inferring an IM model from genetic data, the parameters of interest are demographic parameters of the IM model, denoted as a vector ψ=(θ_1_, *θ*_2_, *θ*_a_, *m*_1_, *m*_2_, *T_S_*). The *i*th locus *D_i_* out of *L* loci are the observations, and the genealogy *G_i_* of *D_i_* is a latent variable that we cannot observe typically (Fig. 2). Fig. 2 depicts the structure of the standard models. The standard models address two levels of uncertainty: the distribution of DNA sequences given genealogy and that of genealogy given an IM model [11,12,25]. We typically assume that there is no recombination within a locus and free recombination between loci. In other words, the *i*th locus *D_i_* out of *L* loci has as its own genealogy *G_i_* and loci are independent. Given genealogy, the genetic data and demography ψ=(θ_1_, *θ*_2_, *θ*_a_, *m*_1_, *m*_2_, *T_S_*) are assumed to be conditionally independent. As the distribution of DNA sequences pDiGi, diverse mutation or substitution models have been developed: infinite-site model [28], JC 69 model [29], HKY model [30], and GTR [31]. There are several useful methods for substitution model selection [25]. A coalescent process [32-34] is a well-known stochastic process for pGiψ, the distribution of genealogy given a species tree or a demographic model. Most methods, including IMa3, MIST, and AIM, are based on this coalescent process. Based on this standard model structure, the likelihood function of ψ is built as follows:

(1) Lψ=∏i=1L∫p(DiGi)p(Giψ)dGi

The likelihood function, so-called Felsenstein’s equation [35], does not have a general closed-form and is difficult to numerically evaluate [3].

## MCMC Simulation and the Mixing Problem

A feasible way to numerically evaluate the likelihood function (Eq. 1) is an MCMC simulation. Extensive reviews of fundamental concepts, diverse algorithms, and MCMC diagnosis are available elsewhere [23,25,36,37]. With a prior distribution on ψ, the posterior density of ψ given data is

(2) pψD∝pΨLΨ=pΨ∏i=1L∫pDiGipGiψdGi

The target density of an MCMC simulation is pψ,G1,…,GLD∝pΨ∏i=1LpDiGipGiψ, and a typical algorithm jointly simulatesnsamples from the target density: ψ1, G11,…,GL1,ψ2, G12,…,GL2,…,ψn, G1n,…,GLn~Pψ, G1,…,GLD.

One of the benefits of such a simulation is an easy approximation of the marginal posterior density (Eq. 2) by making use of simulated values for the parameter of interest. For example, $\psi_{}^{1},\ldots \psi_{}^{n}$, from the jointly simulated values $\left(\psi^{1},\;G_{1}^{1},\ldots ,G_{L}^{1}\right),\left(\psi^{2},\;G_{1}^{2},\ldots ,G_{L}^{2}\right),\ldots ,\left(\psi^{n},\;G_{1}^{n},\ldots ,G_{L}^{n}\right)$, approximately follow pψD in Eq. (2) [38].

**Fig. 3.**
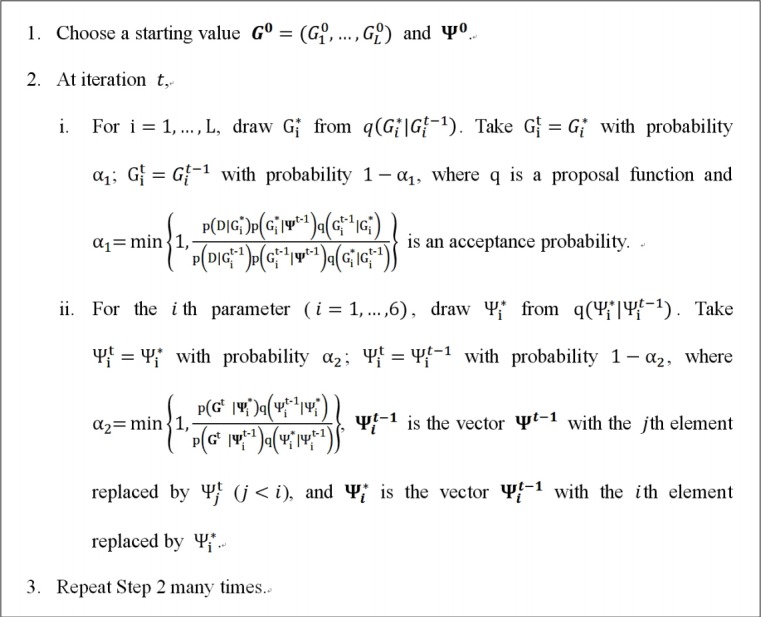
A typical Metropolis-Hastings within Gibbs sampling algorithm.

**Fig. 4.**
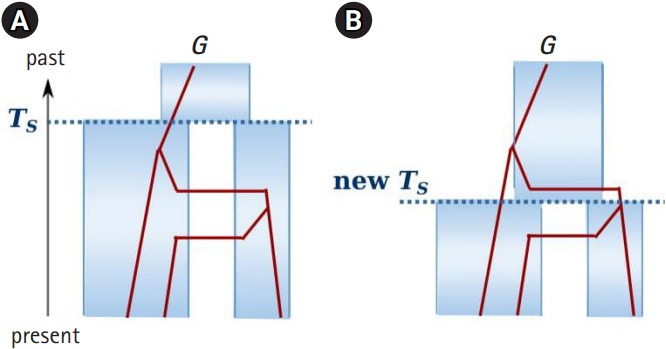
An example of an Markov chain Monte Carlo step to update the splitting time T_S_. (A) The current state of genealogy and all demographic parameters, including T_S_. (B) A newly proposed splitting new T_S_, which is not compatible with the state of genealogy.

A popular MCMC algorithm is a Metropolis-Hastings within Gibbs sampling algorithm (Fig. 3). Within each iteration, all demographic parameters and genealogies are sequentially simulated. For example, Fig. 4A shows the state of the (*t*-1)th iteration for the genealogy of one locus and all demographic parameters ψ including splitting time $T_{S}^{t-1}$. If we try to update the splitting time at the *t*th iteration, we propose a new splitting time $T_{S}^{*}$ using a proposal function *q* and either accept the new value $T_{S}^{t}=T_{S}^{*}$ with probability α=min{1,p(Gtψ*)q(TSt-1TS*)p(Gtψt-1)q(TS*TSt-1)} or reject the new value and retain the previous state $T_{S}^{t}=T_{S}^{t-1}$ with 1-α, where ψ^*^ and ψ^t-1^ includes $T_{S}^{*}$ and $T_{S}^{t-1}$, respectively.

While samples via a traditional Monte Carlo method are independent, MCMC samplers generate autocorrelated draws because the current value is either a different value or the same as the previous. Strong autocorrelations slow down traversing the posterior space and take longer to produce independent-like samples ψ^t^, … ψ^n^ ~ p(ψD) [23,25]. This phenomenon is called a poor mixing of a Markov chain. Mixing issues affect the efficiency, and hence the computing time of an MCMC simulation. In the inference of IM models, poor mixing is a major roadblock to the analysis of genomic data or the co-inference of phylogeny [1,16]. For example, the state of genealogy and demographic parameters are given as Fig. 4A. If a new splitting time proposed at the next iteration is not compatible with the state of genealogy (Fig. 4B), then pGtψ*=0 and the acceptance probability is zero. Therefore, the newly proposed value is automatically rejected, and the previous state should be sampled until a compatible value is proposed. In other words, the acceptance rate of the splitting time is governed by the state of genealogies and can be very small if a lot of loci are considered.

## Inference Methods

### IMa3

The software series of IM/IMa were developed to infer IM models (Table 1) [39,40]. The first software, called IM, analyzes either a single locus [4] or multiple loci [2], and implements MCMC approaches to infer six demographic parameters $\Psi =(\theta_{1},\theta_{2},\theta_{a},m_{1},m_{2},T_{S})$ of an IM model. In other words, the IM software simulates ψ,G1,…,GL~Pψ,G1,…,GLD. Software IMa and IMa2 implement [3]. They simulate values of splitting time and genealogies TS,G1,…,GL~pTS,G1,…,GLD∝∏i=1Lp(DiGi)p(G1,…,GLTS)pTS, but not population sizes and migration rate. It can be done by analytical integration of population sizes and migration rates:

(3) p(G1,…,GLTS)=∫⋯∫p(θ1)p(θ2)p(θa)p(m1)p(m2)∏i=1LpGiψdθ1dθ2dθadm1dm2

This yields a better mixing than software IM by reducing the number of parameters to sample, but it does not resolve the fundamental barrier of the relation between genealogies and splitting time. As a result of an MCMC simulation, the sampled values approximate the marginal posterior of the splitting time: TS1,…,TSn~pTSD. Then IMa2 provides the posterior mean and the maximum *a posteriori* (MAP) estimate with highest posterior density intervals of the splitting time based on the marginal posterior density p(TSD). To infer population sizes and migration rates, IMa and IMa2 do not simulate those parameter values, but directly approximate the marginal densities, p(θiD) for i=1, 2, a and p(miD) for i=1, 2, from sampled values of (T_S_, G_1_, ... , G_L_). The approximated densities p(miD) are employed to perform likelihood ratio tests (LRTs) from migration rates [3]. Software IMa infers 2-population IM models, but IMa2 extends IMa to infer multiple populations (see Table 1 for IMa2p and IMGui).

The most recent version called IMa3 modified the MCMC procedure of IMa2 to infer an IM model parameters as well as phylogeny [16], while IMa2 requires the phylogeny of multiple populations to be known. It is very difficult to co-estimate the phylogeny and IM model parameters, because sampling phylogeny together with IM parameters and genealogies also yields poor mixing. For example, a newly proposed phylogeny may not be compatible with the current state of migrations and is therefore rejected. IMa3 introduces pseudo-migrations, called “*hidden migrations*,” that occurred earlier than the splitting time so that a newly proposed splitting time or phylogeny is not instantly rejected but evaluated with non-zero acceptance probability. For example, if a newly proposed splitting time is younger than existing migrations (Fig. 4B), the migration paths older than splitting time are considered hidden migration paths (*M*_H_) and the genealogy is the one without hidden migrations and compatible with the new splitting time. In other words, the current genealogy, given the new splitting time, is a so-called “*hidden genealogy*” *G_H_=(G, M_H_)*. Given phylogeny τ and demographic parameters ψ, the distribution of the hidden genealogy is partitioned into those of hidden migrations and the genealogy without hidden migrations: p(GHψ,τ)=p(Gψ,τ)pmHψ,τ. Therefore, in the presence of incompatible migration paths, a newly proposed splitting time or phylogeny is not automatically rejected. As a result, IMa3 simulates phylogeny, splitting times and hidden genealogies: τ1,TS1,GH,11,…,GH,L1,τ2,TS2,GH,12,…,GH,L2,…,τn,TSn,GH,1n,…,GH,Ln~Pτ,TS,GH,1,…,GH,LD∝pτpψ∏i=1LpDiGipGiψ,τpmH,iψ,τ

Then τ^1^, ... , τ^n^ from the MCMC samples approximately follow the marginal posterior p(τD). Similar to IMa2, demographic parameters are estimated based on their approximated marginal posteriors.

### MIST

Software MIST [1] implements a 2-step analysis. First, it simulates genealogies without migrations (so-called *coalescent trees* λ___) via an MCMC simulation. Note that no information about a demographic model is necessary in the first step, which alleviates the mixing problem. Second, the joint posterior density p(ψD) in Eq. (2) is approximated from the sampled coalescent trees, and the MAP estimations of all demographic parameters are found.

Although MIST does not sample migrations and the underlying demographic model in step 1, the same posterior density p(ψD) in Eq. (2) is inferred. It is done by separating migration paths from genealogies and applying the importance sampling [38]. The separation of migration paths enables the analytical computation of the density of a coalescent tree:

(4) pλiψ=∫pGiψdMi

where the ith genealogy G_i_=(*λ_i_, M_i_*)and *M_i_* is the set of all migration information. This rewrites Eq. (2) as follows:

(5) pψD∝pψ∏i=1L∫pDiλipλiψdλi

The exact computation of pλiψ employs a continuous time Markov chain representation [1]. In order to reduce the computational burden of the numerical integration in Eq. (5) pλiψ by an MCMC simulation, the importance sampling method was employed. That is, MCMC samplers simulate coalescent trees from posterior $\tilde{p}(\lambda_{i}│D_{i})\propto p(D_{i}│\lambda_{i})\tilde{p}(\lambda_{i})$ rather than pλiψ, where and $\tilde{p}\left(\lambda \right)$ is a flat prior. This MCMC simulation in step 1 does not use any information from the underlying IM model. The use of p~λiDi rather than pλiψ is compensated later in step 2 when the joint posterior density is approximated:

(6) p(ψD)∝pψ∑k=1Lpλkψ

As a result, MIST provides the MAP of all demographic parameters that maximize the joint posterior Eq. (6).

MIST has several strengths statistically and computationally. First, the computational complexity linearly increases with the number of loci. Analyses of thousands of loci do not give rise to mixing problems. Second, similar to IMa series, the approximate p(ψ│D) in Eq. (6) can be used for LRTs for migration rates. While the IM/IMa series uses the marginal

densities, MIST provides the joint distribution of all demographic parameters (Table 2). Since the estimations of demographic parameters are correlated, LRTs based on joint distributions have false-positive rates close to the expected value (e.g., 5%), even when very high false-positive rate occurred by LRTs based on marginal distributions [1,41,42]. Third, the importance sampling method enhances the computational efficiency for model comparisons. When different demographic models are compared, the simulated values from an MCMC simulation in step 1 can be repeatedly employed to infer different demographic models in step 2.

### AIM

AIM [14] implements a Bayesian inference of phylogeny and IM models in using the BEAST platform [15,43]. BEAST is a software platform for phylogenetic analyses, phylodynamics, and population genetics. starBEAST2 [44], an extended BEAST package, was added to estimate species trees in the absence of gene flow. AIM was recently added to estimate the posterior density pψD in Eq. (2) and pτD, like IMa3. Similar to Chung and Hey [1], Müller et al. [22] drived a formula to compute the density of a coalescent tree pλiψ in Eq. (4) and additionally proposed approximations for a fast calculation. One approximation assumes the independence of lineages of the coalescent tree λ: PtL1=l1,L2=l2λ,ψ≈PtL1=l1λ,ψPtL2=l2λ,ψ,

where *L_1_* and *L_2_* are lineages of *λ* at time *t*. AIM implements this independence approximation rather than the exact density pλiψ in Eq. (4).

AIM reparamerized migration rates as follows: migration rate between populations A and B, $m_{A,B}=\alpha_{A,B}\frac{m_{tot}}{\delta_{AB}}$, where α_A,B_ is a scaler that is estimated between every pair of coexisting populations/species, *δ_AB_* is the time to the most recent common ancestor from populations A and B coexisted, and *m_tot_* is an estimated migration rate that allows for a prior distribution on the magnitude of the migration rate expected. This parameterization allows for smaller migration rates between more distant populations. Furthermore, each scaler *α_A,B_*~Exp(1) and all scalers are assumed to be independent. AIM is able to use the priors previously implemented for species tree estimation in starBEAST2 [44].

AIM performs tests for migration rates based on Bayes factors (BFs) [14], while IMa3 and MIST use LRTs (Table 2). A BF as the ratio of marginal likelihoods [37] is wildely used for model selection. Since AIM is a package in the BEAST platform, users can take advantage of other existing packages and MCMC diagnostic tools. However, most packages in BEAST were developed independently [15,45]. Therefore, the results provided by different packages are not connected, and users need to be aware of the different terminologies by each package [15].

## Discussion

IMa3, MIST, and AIM are advanced software that estimate demographic parameters of IM models. IMa3 and AIM sample population tree topologies and all or partial demographic parameters through an MCMC simulation. Therefore, their estimations are based on the marginal posterior distribution of parameters. MIST can estimate the joint posterior distribution of all parameters, thereby providing a joint estimation. IMa3 and AIM estimate population tree topology and migration rates, but their scalability to genomic data is limited or has not been yet examined. MIST scales well with genomic data and can be extended to infer population tree topologies. However, the software currently supports a joint estimation of demographic parameters of 2-population IM models.

While AIM uses BFs for migration rate test, IMa3 and MIST suggest LRTs. While IMa3 compares marginal posterior distributions, MIST provides joint posterior distributions for LRTs. When splitting times are recent, it is important to consider using joint distributions for LRTs in order to avoid a high false-positive [1,41,42].

Long-standing barriers to inferring IM models have been resolved by IMa3, MIST, and AIM. MIST can analyze genome-scale data without sever mixing problems in an MCMC simulation. IMa3 and AIM are able to estimate IM models and phylogeny in the presence of migrations. Nonetheless, there are still unresolved questions and no software implementing sophisticated models to answer the questions. One of the major interests for the future is to relax the strong assumption of constant migration rates and population sizes over time. Current methods that attempt to solve this problem are limited to small data or not capable of inferring IM models from real genetic data analysis [46,47].

## Figures and Tables

**Table 1.** Comparison of Bayesian software MIST, AIM, and IMa3 (IM/IMa series)

Software	No. pop. to analyze	Inference method				Reference
		*θ*'s and *m*’s	T_s_	G^a^	τ	
MIST	2	Density approx.^b^	Density approx.^b^	MCMC	No	[1]
AIM	2 or more	MCMC	MCMC	MCMC	MCMC	[14,22]
IMa3	2 or more	Density approx.^c^	MCMC	MCMC^d^	MCMC	[16]
IM	2	MCMC	MCMC	MCMC	No	[2,4]
IMa	2	Density approx.^c^	MCMC	MCMC	No	[3]
IMa2^e^	2 or more	Density approx.^c^	MCMC	MCMC	No	[3,17]

MIST, AIM, and IMa3 are compared in terms of the number of populations to analyze and inference methods by indicating what Markov chain Monte Carlo (MCMC) samples and which parameters’ posterior densities are approximated rather than sampled. A similar comparison is made with IM/IMa series.

a All methods in this table sample genealogies and other mutation/substitution parameters from MCMC;

b The joint posterior density of the 6 demographic parameters is approximated using MCMC samples;

c The marginal posterior densities of the parameters are approximated using MCMC samples;

d Hidden genealogies are sampled;

e Variants: IMa2p [39] for parallel computation, IMGui [40] for any desktop OS.

**Table 2.** Prior assumptions, migration rate tests and scalability of Bayesian software MIST, IMa3, and AIM

		MIST	IMa3	AIM
Priors	*θ*’s,	Uniform	Uniform	Log-normal [44]
	*m*’s	Uniform	Uniform	Exponetial^a^
	T_S_	Uniform	Uniform	Various [44]
	τ		Uniform	Various [44]
Tests for	m’s	LRT^b^	LRT^c^	Bayes factor
Scalability^d^	Loci	Many (≤10K [1])	Moderate (≤200 [16])	Moderate (≤50 [14])
	Sequences	Few (≤8 [1])	Moderate (≤40 [16])	Moderate (≤133 [22])

a The prior for migration scalers;

b Likelihood ratio test (LRT) compares the joint densities of the 6 demographic parameters;

c LRT compares the marginal densities of migration rates;

d The numbers of loci and sequences in the parentheses are collected from the cited studies. The scalability can depend on computer specifications as well.
